# Overcoming HSP27-mediated resistance by altered dimerization of HSP27 using small molecules

**DOI:** 10.18632/oncotarget.10629

**Published:** 2016-07-16

**Authors:** Jee Hye Kim, Ye Jin Jung, Byeol Choi, Na Lim Lee, Hae Jun Lee, Soo Yeon Kwak, Youngjoo Kwon, Younghwa Na, Yun-Sil Lee

**Affiliations:** ^1^ Graduate School of Pharmaceutical Sciences, Ewha Womans University, Seoul, 120-720, Korea; ^2^ Division of Radiation Effects, Korea Institute of Radiological and Medical Sciences, Seoul, 139-706, Korea; ^3^ College of Pharmacy, CHA University, Pocheon, 487-010, Korea

**Keywords:** HSP27 inhibition, altered dimerization, overcoming resistance, combination therapy

## Abstract

Heat shock protein 27 (HSP27, HSPB1) is an anti-apoptotic protein characterized for its tumorigenic and metastatic properties, and now referenced as a major therapeutic target in many types of cancer. The biochemical properties of HSP27 rely on a structural oligomeric and dynamic organization that is important for its chaperone activity. Down-regulation by small interfering RNA or inhibition with a dominant-negative mutant efficiently counteracts the anti-apoptotic and protective properties of HSP27. However, unlike other HSPs such as HSP90 and HSP70, small molecule approaches for neutralization of HSP27 are not well established because of the absence of an ATP binding domain. Previously, we found that a small molecule, zerumbone (ZER), induced altered dimerization of HSP27 by cross linking the cysteine residues required to build a large oligomer, led to sensitization in combination with radiation. In this study, we identified another small molecule, a xanthone compound, more capable of altering dimeric HSP27 than ZER and yielding sensitization in human lung cancer cells when combined with HSP90 inhibitors or standard anticancer modalities such as irradiation and cytotoxic anticancer drugs. Therefore, altered dimerization of HSP27 represents a good strategy for anticancer therapy in HSP27-overexpressing cancer cells.

## INTRODUCTION

Molecular chaperones, including heat shock proteins (HSPs), help tumor cells cope with stress-induced misfolded proteins and play prominent roles in cellular signaling and transcriptional regulatory networks. HSP27 (HSP27 [human form] and HSP25 [murine form]) has been identified as a critical mediator in cancer progression, preventing apoptosis in transformed cells [1–4]. In a wide range of human cancers, increased levels of HSP27 are closely associated with tumorigenesis, metastasis, resistance to anticancer therapeutics, and, accordingly, poor prognosis [4, 5]. Therefore, HSP27 inhibition as an adjuvant of radio- and chemo-therapy has clinical implications. HSP27 gene silencing by OGX-427, a second-generation antisense oligonucleotide, induces sensitization in radio- and chemo-resistant cancer cells [6]. Moreover, OGX-427 overcomes treatment resistance by HSP90 inhibitors, including natural compounds such as geldanamycin and its analog 17-allylamino-17-demethoxy-geldanamycin (17-AAG), or synthetic compounds including PF-04928473, due to compensatory mechanisms involving activation of HSP27 [7, 8]. Accordingly, HSP27 inhibition is an attractive therapeutic target.

Although an appealing cancer target, HSP27 acts through an ATP-independent mechanism and is therefore not susceptible to inhibition by 17-AAG–derived small molecules. Recently, a small molecule triazole ribonucleoside was reported to induce apoptosis in pancreatic cancer cells by decreasing HSP27 levels; however the exact mechanism and the specificity of the drug against HSP27 knockdown remain unclear [4, 9–11]. Aside from this compound, no small molecules have been developed as HSP27 inhibitors for cancer therapy, although functional HSP27 inhibition may be a good strategy for combination therapy with HSP90 inhibitors, chemotherapeutic agents, or radiation. Strategies to inhibit HSP27 at the mRNA level offer an alternative approach to inhibiting targets, including short interfering RNA (siRNA) [12, 13] or antisense oligonucleotides such as OGX-427 to suppress target levels [14]. While siRNA can potently and specifically inhibit target genes *in vitro*, cellular uptake and activity *in vivo* require drug delivery systems that have proven challenging and rate-limiting.

Another interesting approach to targeted inhibition of HSP27 involves the use of HSP27 peptides that interact with HSP27 and promote apoptosis induced by chemotherapeutics, similar to HSP27 silencing [15–19]. However, to make these peptides stable for *in vivo* and therapeutic use, further processing is needed, such as conjugation with PEG to increase molecular mass and extend the half-life of peptides by slowing renal filtration [20].

We previously demonstrated that zerumbone (ZER), a cytotoxic component isolated from a natural product, *Zingiber zerumbet Smith*, induced cross-linking of the HSP27 protein by insertion between the disulfide bonds of HSP27. ZER-mediated altered cross-linking of HSP27 modified normal HSP27 dimerization, which resulted in a sensitizing effect to tumors after treatment with radiation (IR). Therefore, altered cross-linking by ZER was suggested as a novel strategy for inhibition of HSP27-mediated resistance [21]. In this study, we identified a more potent HSP27 cross linker, SW15, a synthetic xanthone compound which yielded stronger sensitization, when it was combined with HSP90 inhibitors. Moreover, SW15 also showed potent synergism with conventional anticancer modalities.

## RESULTS

### The same xanthone moiety with different side chains caused different cross linking activity of HSP27

**Figure 1:**
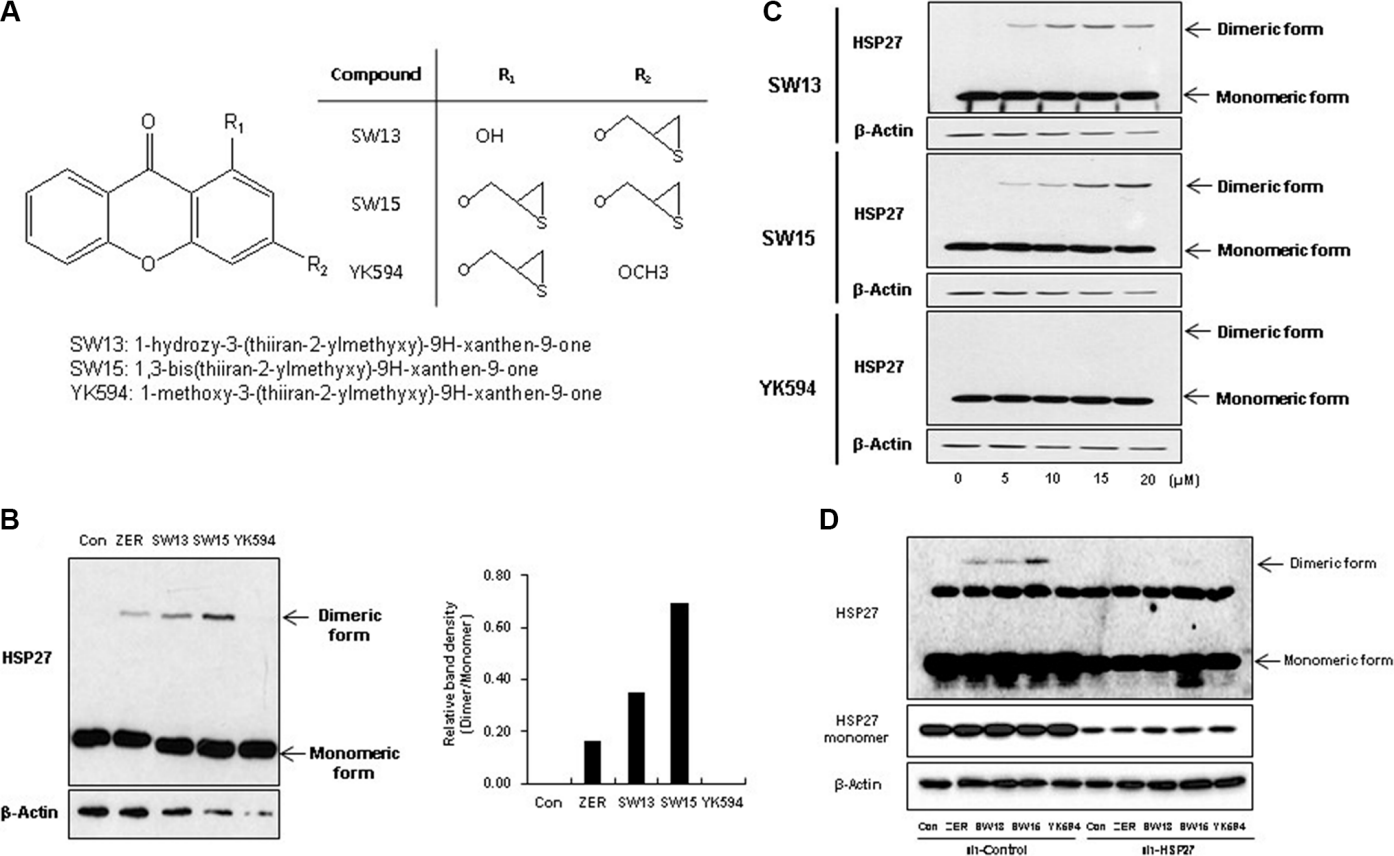
Xanthone structures led to different altered cross linking activity of HSP27 (**A**) Structures of xanthone compounds (SW13, SW15 and YK594). (**B**) Effects of the three xanthone compounds on altered cross-linking of HSP27. NCI-H460 cells were treated with each compound (12.5 μM for 3 h), and cell lysates were analyzed by Western blotting (left). Relative protein band intensity was calculated by comparing densitometric scans of the sample immunoblots with the values of control samples set at 1 (right). (**C**) NCI-H460 cells were treated with each compound (0, 5, 10, or 20 μM for 12 h), and cell lysates were analyzed by Western blotting. (**D**) NCI-H460 cells stably transfected with control (sh-Control) or shRNA of HSP27 (sh-HSP27) were treated with each compound (10 μM for 12 h), and cell lysates were analyzed by Western blotting. Zerumbone (ZER, 10 μM) was used for a positive control.

ZER promotes cross-linking of HSP27 to form altered dimers, inhibiting oligomerization of HSP27. Because ZER is a naturally occurring compound, large-scale production has limitations. Moreover, chemical characteristics of ZER are not druggable, because of low solubility and limitation of mass production. During large scale screening of HSP27 cross-linking activity using synthetic compounds (Supplementary Figure S1A), we found that xanthone moiety compounds yielded different HSP27 cross-linking activity, with variation in potency according to side chain structures (Supplementary Figure S1B). Next, we selected 3 xanthone compounds with differing side chain structures, SW13, SW15, and YK594, because they promoted different amounts of cross-linking of HSP27. SW15 displayed the strongest cross-linking activity, SW13 had less effect than SW15, and YK594 showed no cross linking activity (Figure 1A, 1B and 1C). The cytotoxic activitiy of SW13. SW15 or YK594 was weak, with IC50 values in NCI-H460 cells of > 80, 23.87 ± 1.83 and > 80 uM, respectively, compared to taxol (Supplementary Figure S2). Moreover, HSP27 cross-linking activity disappeared when shRNA of HSP27 was stably transfected (Figure 1D), suggesting that altered cross- linking of HSP27 was HSP27-dependent. The next experiments were performed using SW15, which yielded the highest cross-linking of HSP27. To elucidate whether cross-linking of HSP27 by SW15 was specific to the HSP27 protein, we evaluated cross-linking activity of other proteins with cysteine residues that form disulfide bonds. Proteins of HSP90, HSP70, NFκB, JNK2, Akt1 and β-Actin did not form cross-linked dimer forms after treatment of SW15 (Supplementary Figure S3A). The analysis of mass spectrophotometry for the dimerized form of HSP25 (HSBP1, murine form) at approximately 50 kDa revealed that this protein was the cross-linking form of HSP25 (Supplementary Figure S3B).

### The HSP27 cysteine residue is important for altered cross linking of HSP27 through the xanthone compound

**Figure 2:**
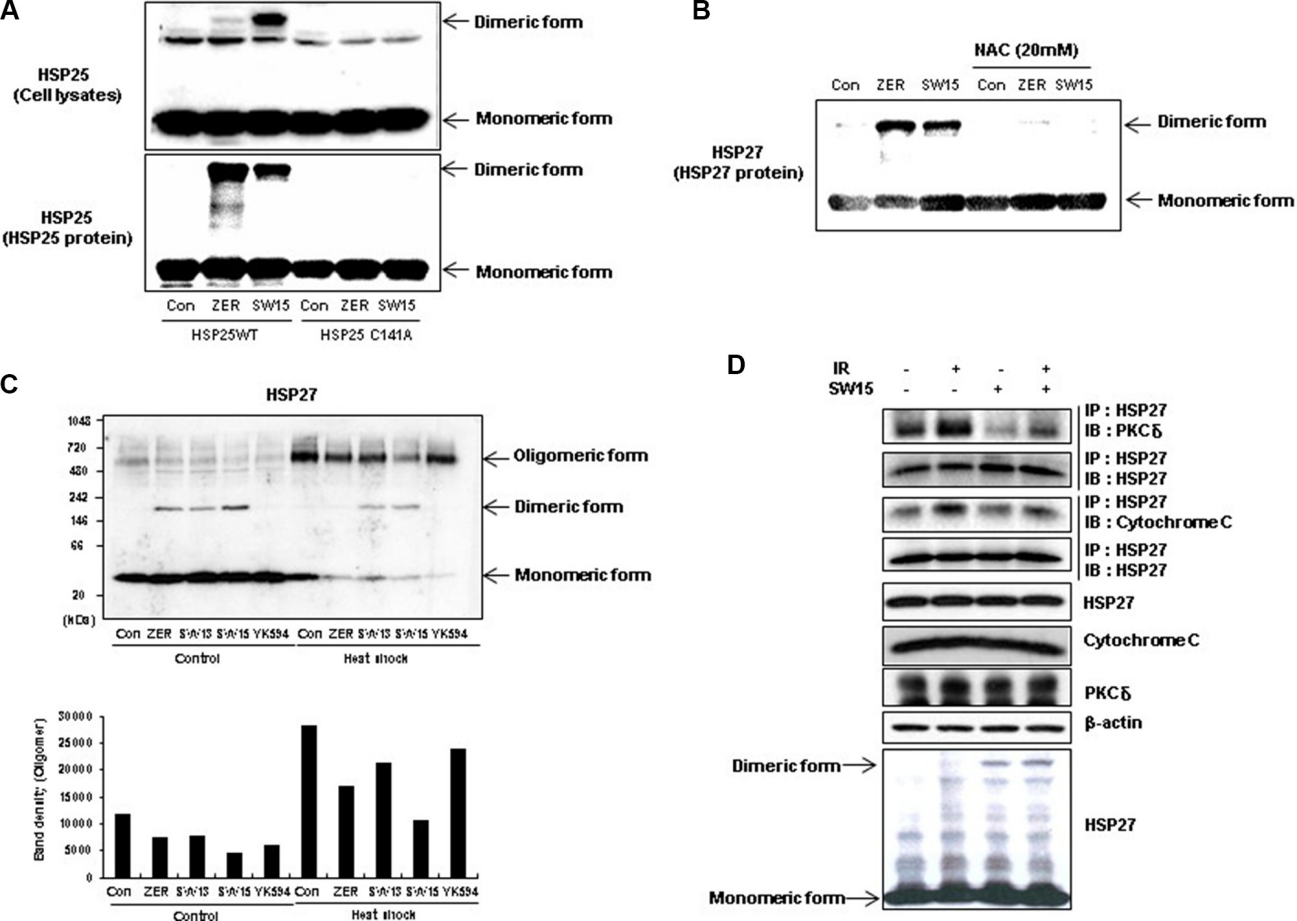
Altered cross linking by a xanthone compound occurred in the cysteine residue of HSP27 and inhibited HSP27 oligomerization (**A**) NCI-H460 cells were transfected with wild type HSP25 (WT) or dimerization-deficient mutant HSP25 on the cysteine residue (C141A) and cross-linking of the exogenous HSP25 was analyzed by SDS-PAGE (upper). Murine recombinant wild type HSP25 protein (WT) or dimerization-deficient mutant HSP25 protein (C141A) after treatment with the compounds (0.5 mM for 3 h), was also analyzed by SDS-PAGE (bottom). (**B**) NCI-H460 cells were treated with DMSO, ZER, or SW15 (10 μM) for 12 h with or without treatment with 20 mM N-acetyl cysteine (NAC) and cross-linking of exogenous HSP25 was analyzed by SDS-PAGE. (**C**) NCI-H460 cells were treated with compounds (ZER, SW13, SW15 or YK594, 10 uM for 12 h) with or without heat shock (43°C for 90 min, and further incubation at 37°C for 0 and 12 h), and cross-linking of HSP27 was analyzed in non-reducing (without reducing agents and boiling) and reducing conditions (with reducing agents and boiling). Relative protein band intensity of oligomerized HSP27 was calculated by comparing densitometric scans of the sample immunoblots with the values of control samples set at 1. Zerumbone (ZER, 10 μM) was used for a positive control. (**D**) NCI-H460 cells were treated with SW15 (10 μM) with or without IR (10 Gy). After 12 h of irradiation, interactions of HSP27 with cytochrome c or PKCdelta were analyzed by immunoprecipitation (IP).

ZER-mediated altered cross linking occurs in a specific cysteine residue (position 141 in mice and 137 in humans), and so we examined whether SW15 also directly affected cross-linking of HSP27 at the same cysteine residue. The mutant form of HSP25 (we transfected the murine form of HSP25 to differentiate the expression of endogenous human HSP27), which replaces cysteine at position 141 with alanine (HSP25-C141A) and does not induce cross-linking of HSP25, was transfected. SW15 induced cross-linking of HSP25-WT, but not HSP25-C141A (Figure 2A). Moreover, pretreatment with N-acetyl cystein (NAC), a thiol reducing agent caused SW15-mediated altered cross linking of HSP27 to disappear, suggesting that the cysteine residue of HSP27 is important for SW15-mediated altered cross linking of HSP27 (Figure 2B). Normal dimerization of HSP27 induced large oligomerization of HSP27 when it was detected in non-reducing gels, and not in reducing gels. However, SW15 induced cross-linking of HSP27 that was different from the common disulfide bond and inhibited the formation of normal large-size oligomers of HSP27 (Figure 2C) [21]. To determine whether altered cross-linking of HSP27 could affect binding activity with apoptotic molecules such as cytochrom c [24] or PKCdelta [17, 18], we pretreated samples with SW15 before IR and binding activity between HSP27 and cytochrome c or PKCdelta was examined at 12 hr after IR. Increased binding activity between HSP27 and cytochrome c or PKCdelta after IR was inhibited by SW15 (Figure 2D). From the data, we conclude that SW15-mediated cross-linking of HSP27 was different than normal HSP27 dimerization and might prevent chaperone activity.

### The combination of the HSP90 inhibitors and the xanthone compound sensitized cancer cells

**Figure 3:**
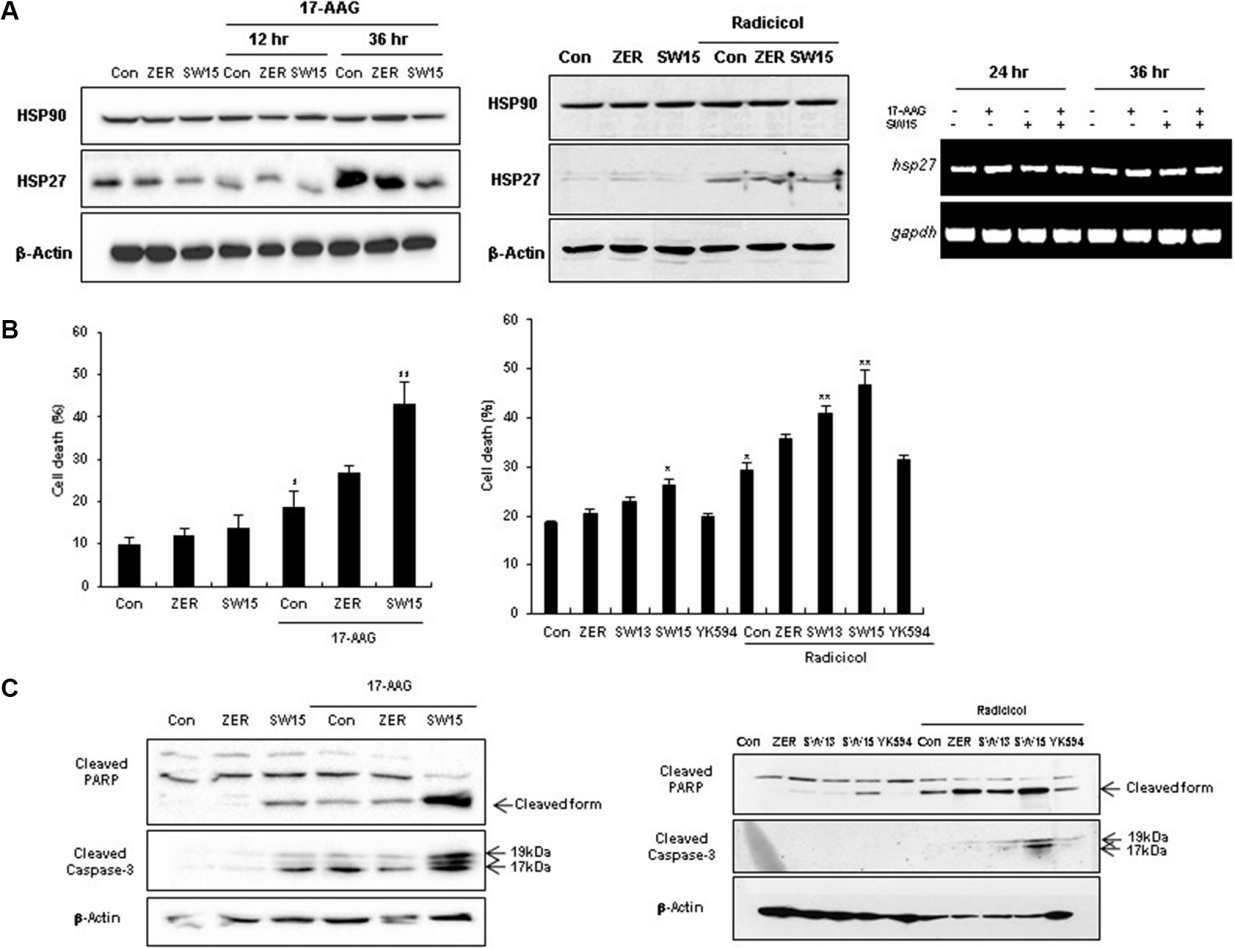
The xanthone compound induced sensitization to cancer cells in combination with HSP90 inhibitors (**A**) NCI-H460 cells were treated with SW15, SW13, YK594 or ZER (10 μM) for 12 and 36 h, with or without 17-AAG (3 μM) (left) or for 24 h, with or without radicicol (1 μM), Western blotting was performed (middle). RT-PCR was performed at 24 and 36 h after SW15 (10 μM) treatment with or without 17-AAG (3 μM) (right). Cell death was analyzed by flow cytometry after PI staining (**B**) and Western blot (**C**) was performed at 24 h after 17-AAG or radicicol treatment. Results are the means and standard deviations of three independent experiments (*p < 0.05 *ver* untreated control and **p < 0.05 *ver* 17-AAG or radiciol alone). Relative band intensity of the cleaved form of proteins was calculated by comparing densitometric scans of the sample immunoblots with the values of control samples set at 1.

**Figure 4:**
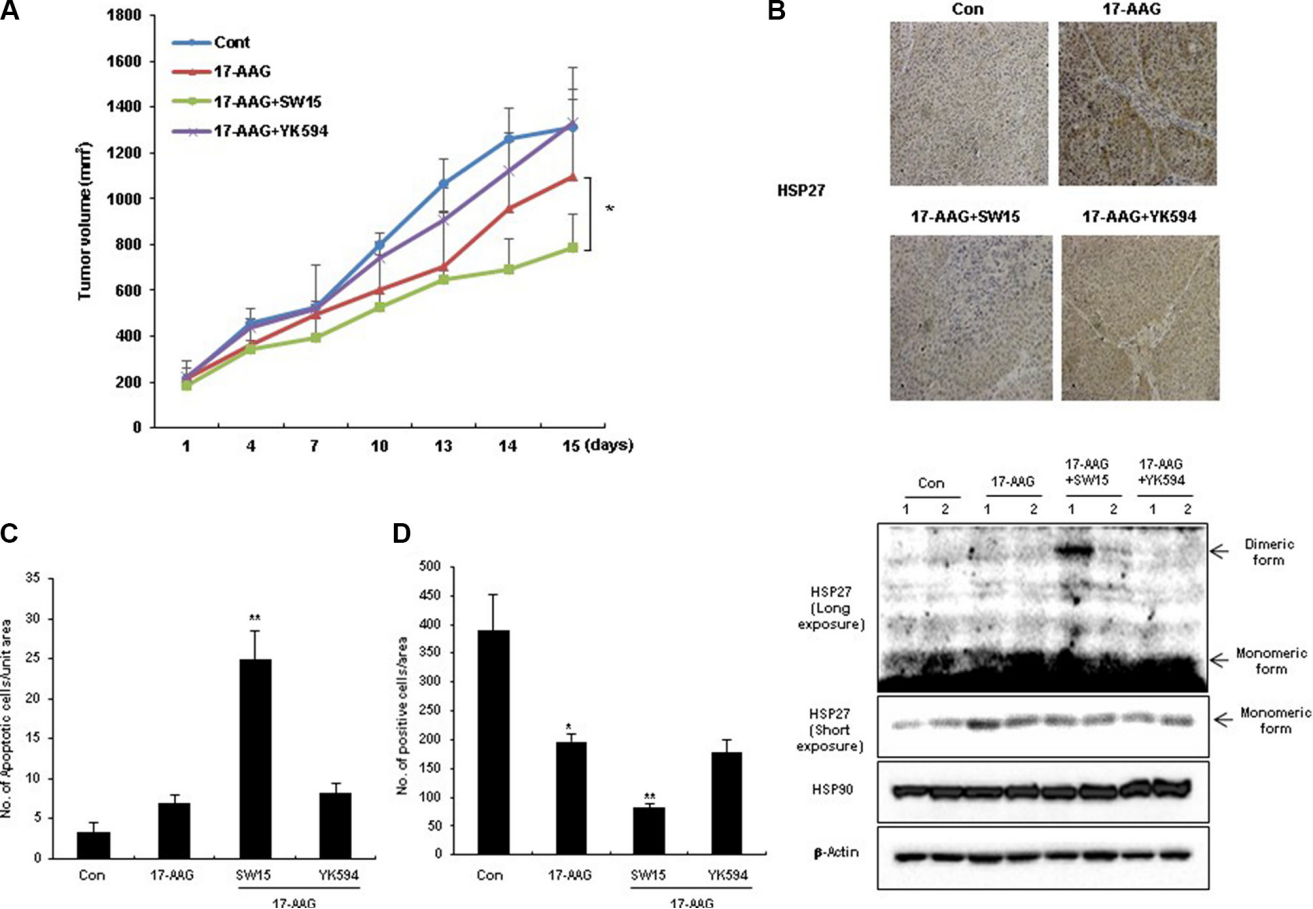
The xanthone compound showed synergistic regression effects to xenografted tumors in combination with HSP90 inhibitors (**A**) NCI-H460 cells were injected subcutaneously into BALB/c nude mice (*n* = 3/group). Xenografted mice were treated 6 times with SW15 or YK594 (6.8 mg/kg per each) delivered with a local regional application in combined with 6 times intraperitoneal treatment of 17-AAG (25 mg/kg). Tumor size was measured twice weekly. Results are the means and standard deviations (*p < 0.05). TUNEL staining (**C**) and Ki-67 staining (**D**) were performed using tumor tissues. Graph represents mean and standard deviation (*p < 0.05 *vs* untreated control group and **p < 0.05 *vs* 17-AAG alone treated group). (**B**) NCI-H460 xenografted nude mice (each group had 2 mice) were treated three times every 2 days with SW15 or YK594 (6.8 mg/kg per each) with or without 17-AAG (25 mg/kg). Three hours after the last treatment, tumor tissues were extracted, and immunohistochemistry for HSP27 was performed (upper). Western blotting analysis for HSP27, HSP90, and β-Actin was also performed (bottom).

Although HSP90 inhibitors offer promise for cancer cell treatment, resistance emerges early due to compensatory mechanisms involving activation of HSF1, which attenuates drug effectiveness [41]. Attenuation is associated with increased expression of HSP27, HSP70, and HSP90, mediating tumor cell survival and treatment resistance [22]. To elucidate whether our xanthone compound could sensitize cancer cells after treatment with the HSP90 inhibitors 17-AAG or radicicol, we first examined the expression of HSP27 after treatment with 17-AAG or radicicol. 17-AAG treatment for 36 hr and radicicol treatment for 24 hr dramatically increased the expression of HSP27 (Figure 3A). Cell death, cleaved casepase-3 and PARP cleavage data suggested that the combination of SW15 with 17-AAG or radicicol synergistically sensitized the lung cancer cells and that the potency was more dominant for SW15 than ZER (Figure 3B and 3C). *In vivo* data using nude mice after grafting of NCI-H460 cells indicated that SW15 led to sensitization in combination with 17-AAG, but YK594, which did not induce any altered dimerization of HSP27, did not (Figure 4A). Increased expression of HSP27 was detected in 17-AAG treated tumor tissues when examined by both immunohistochemistry (Figure 4B, upper) and Western blotting (Figure 4B, bottom). Moreover, cross-linking of HSP27 was only observed in SW15 treated tumor tissues, but not YK594 treated ones (Figure 4B, bottom). Apoptotic and Ki-67-positive areas in tumor tissues also correlated well with the sensitizing effects of SW15 in combination with 17-AAG (Figure 4C, 4D, and Supplementary Figure S4). We also compared anticancer activity between SW15 and RP101, a small molecule HSP27 inhibitor which is under the phase II clinical trial [23] using lung cancer cells xenograft model and found that SW15 showed the anticancer activity in combination with 17-AAG (Supplementary Figure S5). From the data, we concluded that SW15-mediated cross-linking of HSP27 in combination with HSP90 inhibitors has a sensitization effects in lung cancer cells.

### The cysteine residue of HSP27 is important for sensitization of cancer cells by the xanthone compound in combination with HSP90 inhibitor

**Figure 5:**
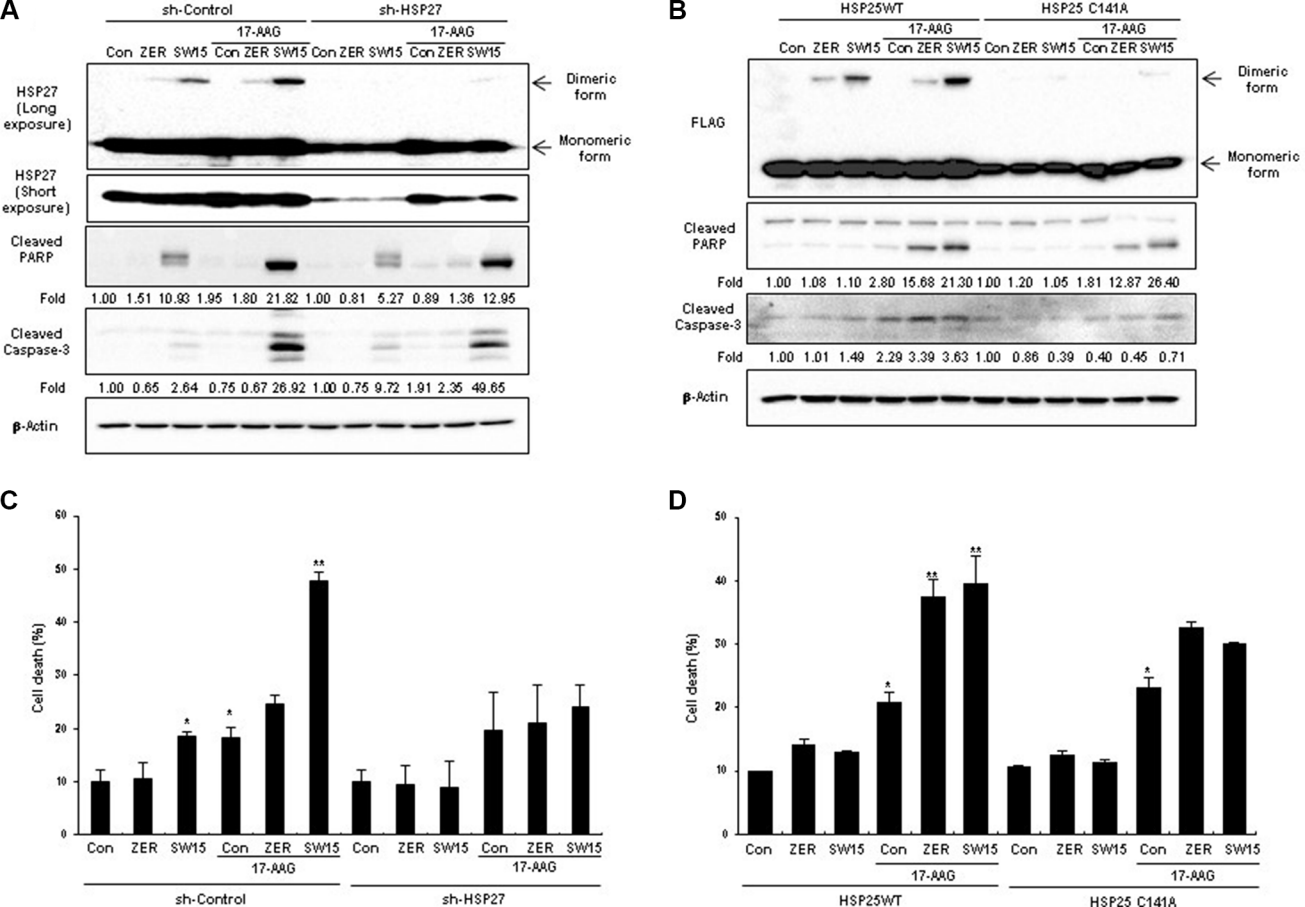
Altered cross linking of HSP27 by a xanthone compound was important for sensitization of cancer cells in combination with an HSP90 inhibitor (**A**) NCI-H460 cells stably transfected with control (sh-Control) or shRNA of HSP27 (sh-HSP27) were treated with SW15 (10 μM for 3 h), and cell lysates were analyzed by Western blotting. Relative protein band intensity of oligomerized HSP27 was calculated by comparing densitometric scans of the sample immunoblots with the values of control samples set at 1 and expressed as a fold increase. (**B**) NCI-H460 cells were transfected with wild type HSP25 (WT) or dimerization-deficient mutant HSP25 (C141A) and cell lysates were analyzed by SDS-PAGE. Relative protein band intensity of oligomerized HSP27 was calculated by comparing densitometric scans of the sample immunoblots with the values of control samples set at 1 and expressed as a fold increase. (**C**), (**D**) NCI-H460 cells were treated with ZER or SW15 (10 μM) for 24 h, and then cell death was analyzed by flow cytometry after PI staining (*p < 0.05 *vs* untreated control cells and **p < 0.05 *vs* corresponding 17-AAG alone treated cells). Zerumbone (ZER, 10 μM) was used for a positive control.

To evaluate whether the synergistic effects of SW15 in combination with HSP90 inhibitors were derived from altered dimerization of the cysteine residue of HSP27, HSP27 knockdown cells and the mutant form of HSP25 at cysteine 141 residue (HSP25-C141A) were treated with 17-AAG and SW15. SW15-induced cross-linking of HSP27 was not seen in HSP27 knockdown cells, as well reduced sensitization effects, when it was detected by cleaved PARP, cleaved caspase 3, and cell death (Figure 5A and 5C). Similarly, HSP25-C141A blocked SW15-mediated cross-linking (Figure 5B and 5D) accompanied by reduced sensitization (Figure 5D), providing further evidence that the cysteine residue of HSP27 is important for SW15-mediated altered cross-linking of HSP27 and sensitization effects in combination with 17-AAG.

### The xanthone compound sensitized cancer cells in combination with radiation

**Figure 6:**
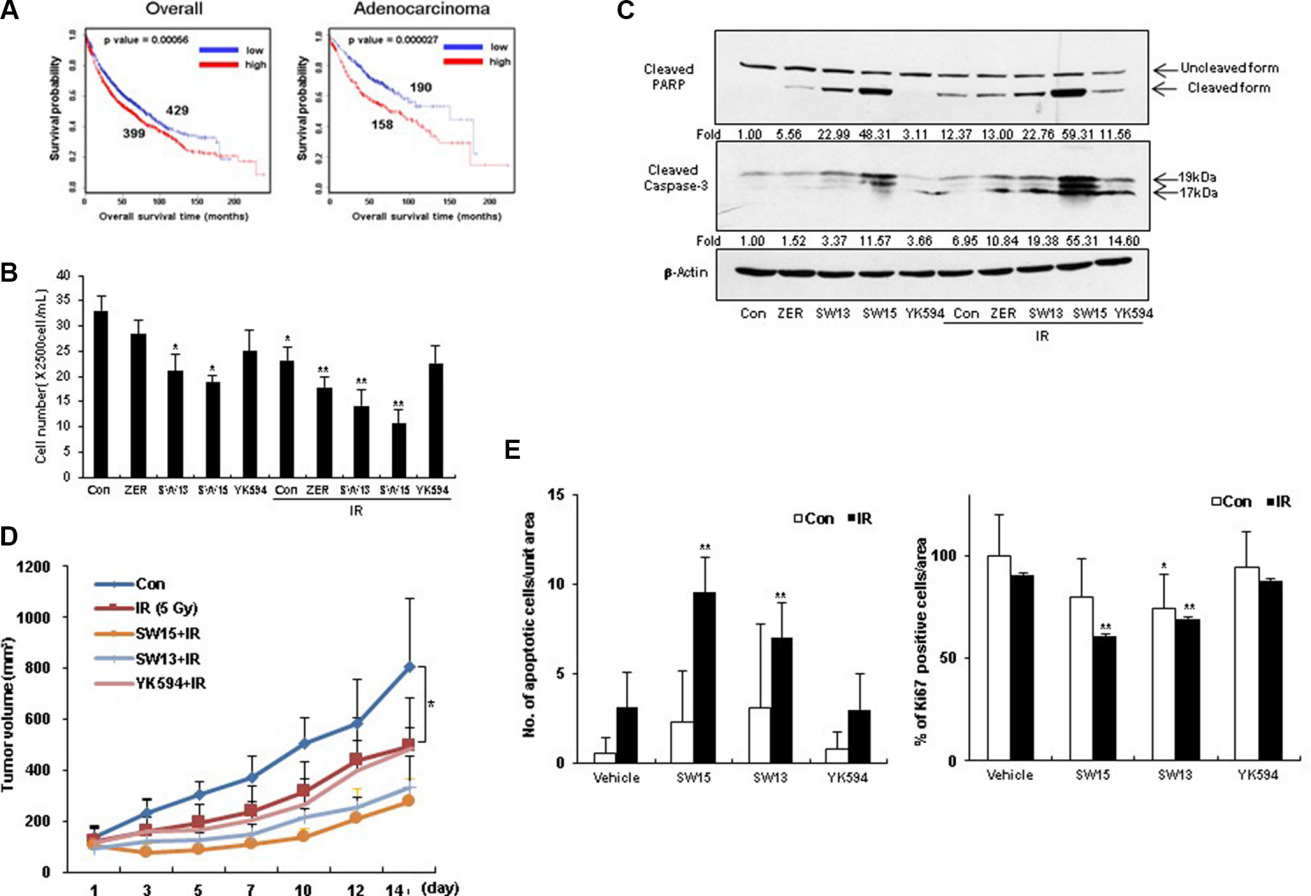
The xanthone compound induced radiosensitization (**A**) Kaplan-Meier (KM) plot for *hsp27* gene in overall lung cancer patients (including adenocarcinoma and squamous cell carcinoma patients) and lung adenocarcinoma patients was shown with *p* value. (**B**) NCI-H460 cells were treated with ZER, SW13, SW15, and YK594 (10 μM) with or without IR (3 Gy). After 24 h of irradiation, cell survival was counted using trypan-blue. Results are the means and standard deviations of three independent experiments (*p < 0.05 *vs* untreated control cells and **p < 0.05 *vs* IR alone treated cells). (**C**) Cleavage of PARP and Caspase-3 was analyzed by Western blot. Relative band intensity of the cleaved form of proteins was calculated by comparing densitometric scans of the sample immunoblots with the values of control samples set at 1 and expressed as a fold increase. Zerumbone (ZER, 10 μM) was used for a positive control. (**D**) NCI-H460 cells were injected subcutaneously into BALB/c nude mice (*n* = 3/group). After 5 Gy irradiation, xenografted mice were treated 6 times with SW15, SW13 or YK594 (6.8 mg/kg) delivered with a local regional application. Tumor size was measured twice weekly. Results are the means and standard deviations (*p < 0.05). (**E**) TUNEL staining (left) and Ki-67 staining (right) were performed using tumor tissues. Graph represents mean and standard deviation (*p < 0.05 *vs* untreated control group and **p < 0.05 *vs* IR alone group).

Because, HSP27 overexpression showed reduced patient survival in lung adenocarcinoma, according to at least one publically available clinicogenomics database (Figure 6A), we next examined whether altered cross-linking of HSP27 by SW15 affected HSP27-mediated resistance after treatment with conventional anticancer modalities. Pretreatment with SW15 for 3 hr before IR decreased cell death as measured by MTT assay, and combined treatment of SW15 with IR significantly increased IR-induced cell death when examined at 24 hr of IR (Figure 6B). In addition, cleavages of caspase 3 and PARP were synergistically increased 3 hr after pretreatment with SW15 before IR when examined 24 hr after IR (Figure 6C). *In vivo* data using nude mice after grafting of NCI-H460 cells indicated that SW15 showed the strongest radio-sensitization effect among the 3 compounds (SW13, SW15 and YK594) in combination with 5 Gy IR, even though the tumor regression effects of these 3 compounds alone without IR were similar (Supplementary Figure S6A). However, unlike SW15, treatment with YK594, which did not induce any altered cross-linking of HSP27, did not result in any sensitization to radiation in the xenografted mice (Figure 6D). Apoptotic and Ki-67-positive areas in tumor tissues also correlated well with the radio-sensitization effects of the three xanthone compounds (Figure 6E and Supplementary Figure S6B). From the data, we conclude that SW15-mediated cross-linking of HSP27 is specific for HSP27 protein and has a radio-sensitization effect in cancer cells.

### The xanthone compound promoted sensitization to cancer cells in combination with the anticancer drugs

**Figure 7:**
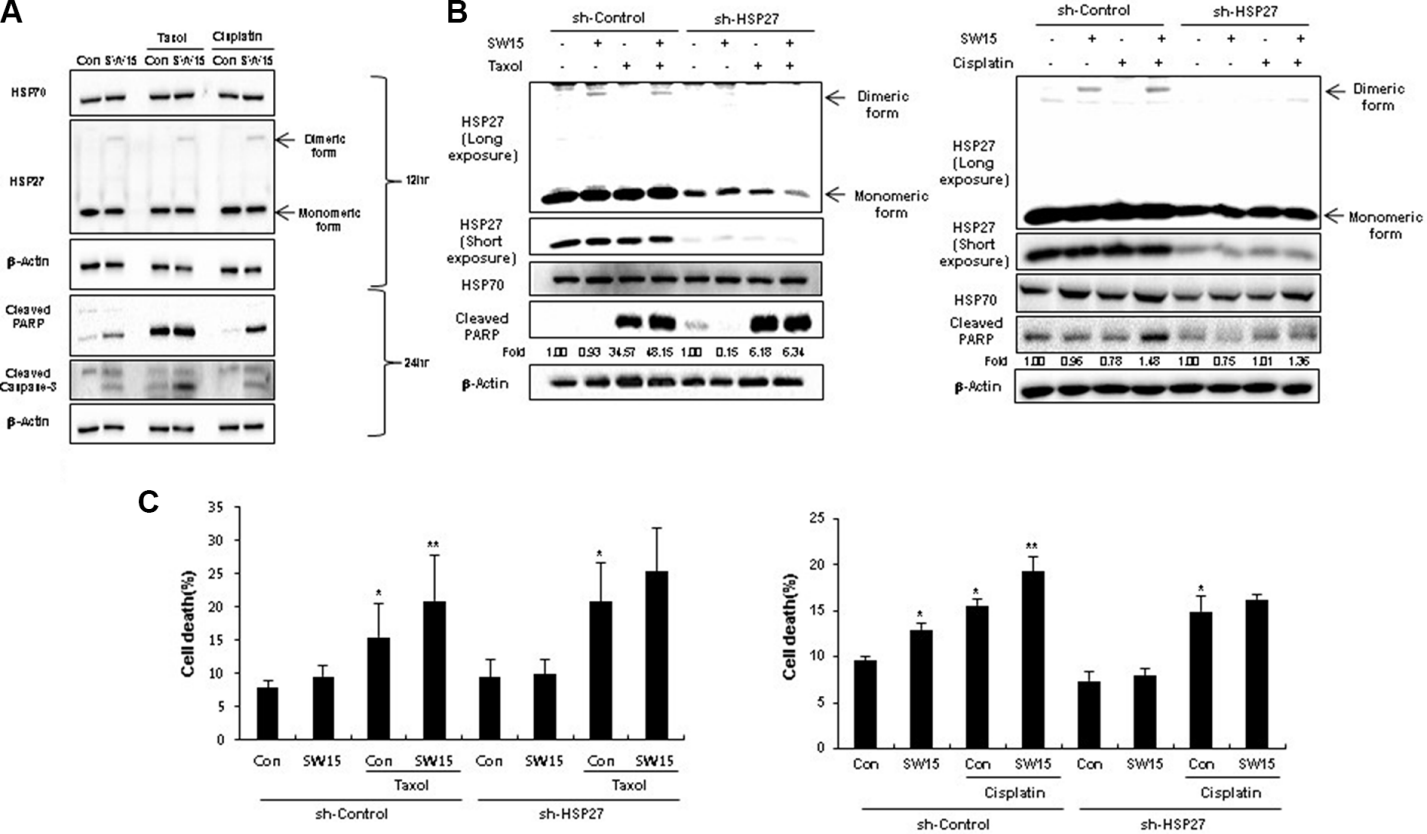
The xanthone compound induced sensitization to cancer cells in combination with conventional anticancer drugs (**A**) NCI-H460 cells were treated with SW15 (10 μM) for 12 and 24 h, with or without cisplatin (3 μM) or Taxol (0.01 μM), and Western blotting was performed. (**B**) NCI-H460 cells stably transfected with control (sh-Control) and shRNA of HSP27 (sh-HSP27) were treated with SW15 (10 μM) for 12 h, with or without cisplatin (20 μM) or Taxol (0.005 μM), and cell lysates were analyzed by Western blotting. Relative band intensity of the cleaved form of proteins was calculated by comparing densitometric scans of the sample immunoblots with the values of control samples set at 1 and expressed as a fold increase. (**C**) Cell death was analyzed by flow cytometry after propidium iodide (PI) staining. Results are the means and standard deviations of three independent experiments (*p < 0.05 *vs* untreated control cells and **p < 0.05 *vs* corresponding taxol or cisplatin alone treated cells).

To elucidate whether SW15-mediated sensitization by altered cross linking of HSP27 was a universal phenomenon, anticancer drugs taxol or cisplatin were combined with SW15. Cell viability, cleaved casepase-3 and PARP cleavage data suggested that combination of SW15 with cisplatin or taxol sensitized the lung cancer cells (Supplementary Figure S7 and Figure 7A). Moreover, cells with stably transfected shHSP27 with reduced SW15-mediated altered cross linking of HSP27 showed less sensitization to taxol or cisplatin in combination with SW15, when it was examined by cleaved PARP production (Figure 7B). Cell death as detected with PI staining also suggested that SW15 potentially increased cell death in combination with taxol or cisplatin in HSP27 overexpressed cells (Figure 7C).

## DISCUSSION

The negative effect of HSP27 is the result of its ability to modulate key steps of the apoptotic cascade through interaction with crucial regulators such as cytochrome c [24, 25], pro-caspase-3 [26, 27], DAXX [28], Akt [29], Stat3 [30], eIF4E [31], PKCdelta [17] and F-actin, an upstream modulator of apoptosis [18]. HSP27 is expressed in many cancers and is known to control resistance against treatment with cytotoxic drugs, development of metastases, and prevention of apoptosis [32]. Expression of HSP27 is upregulated in many cancer cells and is usually associated with a poor clinical outcome [33, 34, 19], making HSP27 a novel druggable target in cancer biology and its inhibition a new strategy in the development of innovative therapeutics [35]. These recent studies suggest the possibility of HSP27 inhibition as a molecular target for cancer therapy. However it is believed that, unlike other HSPs, small HSPs like HSP27 do not bind ATP, making HSP27 a difficult target for small compounds [36, 37].

We previously identified the new small molecule HSP27 inhibitor ZER, which displayed a novel mechanism of altered cross- linking of HSP27, and induced cancer sensitization in combination with IR [21]. In this study, we identified an even stronger cross linker of HSP27, a xanthone compound (SW15), which also yielded greater sensitizing effects than ZER in combination with IR. However, the same xanthone compound with a different side chain (YK594), did not induce any altered cross linking of HSP27 and did not show any sensitization effects, suggesting that some critical structures for insertion to the cysteine residue of HSP27 may be present. In the case of ZER, the unsaturated carbonyl group is important for insertion to the cysteine residue of HSP27 [21], however, all unsaturated carbonyl groups did not consistently induce altered cross linking of HSP27 (data not shown). Because the complete structure of HSP27 is not available, more detailed experiments are necessary to fully elucidate the interaction between small molecules and HSP27.

Cys 141 (the normal dimer site for mouse HSP25, cysteine 137 for human HSP27) is thought to be important for altered cross linking by SW15, and treatment with NAC blocked altered cross linking activity of SW15, providing evidence for a close relationship. In this study, a protein with a molecular weight of approximately 50 kDa was observed after SW15 treatment. Unlike to ZER, we did not detect insertion of complete SW15 structure to cross linked dimer form of HSP27 by mass spectrophotometry, suggesting that chemical changes were made to SW15 for HSP27 cross- linking. When the binding activity of HSP27 with apoptotic molecules was examined after treatment with SW15, increased binding activity between HSP27 and cytochrome c or PKCdelta after IR was inhibited by SW15 treatment. Moreover, cross-linking of HSP27 by SW15 was specific for HSP27 protein, because cross-linking activity of other proteins that have cysteine residues and form disulfide bonds such as HSP90, HSP70, NFkB, JNK2, Akt1, and β-Actin, did not induce cross-linked dimer forms by SW15. From the data, we conclude that SW15-mediated cross-linking of HSP27, which is specific for HSP27 protein, may yield a functionally defective form.

HSP90 interacts with several proteins involved in growth factor receptors, cell cycle regulators, and signaling kinases, including protein kinase B (Akt) or androgen receptor (AR) [39, 40]. Tumor cells express higher HSP90 levels and activity than benign cells [41, 42], and HSP90 inhibition has emerged as a target in cancers. However, HSP90 inhibitors may cause compensatory mechanisms, such as the activation of HSP27 [9]. Therefore, treatment combining an HSP90 inhibitor and an HSP27 inhibitor might be an attractive therapeutic strategy. Indeed, SW15 inhibited HSP27 expression due to altered cross-linking activity after treatment of HSP90 inhibitor. Moreover, combined treatment of HSP90 inhibitor and SW15 synergistically enhanced cancer cell death *in vitro* and *in vivo*, depending on HSP27 expression and the presence of the normal dimerization site at Cys141, suggesting that addition of an HSP27 inhibitor with altered cross linking of HSP27 could overcome HSP90 inhibitor-mediated compensatory mechanisms such as increased expression of HSP27.

Cytotoxic anticancer modalities such as IR, taxol and cisplatin are also involved in HSP27-mediated resistance [43–46], making an HSP27 cross linker a useful strategy for sensitization of cancer cells. Indeed, our results indicated that SW15 in combination with IR, taxol or cisplatin synergistically induced sensitization of lung cancer cells, suggesting that SW15 might be a universal sensitizer to combat HSP27-mediated resistance.

The treatment of SW15 alone did not show any strong cytotoxicity *in vitro* assay, however, *in vivo* data revealed that antitutmor activity of SW15 is similar to the effects of 5 Gy IR, which may give a stronger rationale to SW15 as a candidate of anticancer drug.

In conclusion, since HSP27 expression correlates well with poor survival of lung tumor patients, especially those with lung adenocarcinoma, inhibition of HSP27 represents a good strategy for sensitization of cancer cells in combination with conventional anticancer modalities. Small molecules such as ZER and SW15, which promote altered cross linking of HSP27, offer a promising approach for inhibition of HSP27, although further research is needed to evaluate the safety and efficacy of both compounds in a clinical setting.

## MATERIALS AND METHODS

### Compounds and chemicals

SW13, SW15, and YK594 were synthesized as described in Supplementary information. zerumbone (ZER), was isolated and purified from the dried rhizomes of Zimgiber zerumbet Smith (18). Tanespimycin (17-allylamino-17-demethoxygeldanamycin, 17-AAG) (Selleckch em, S1141) and radicicol (Sigma Aldrich, R2146) was dissolved in DMSO and diluted to 10 mM with tissue culture medium immediately prior to use. Cisplatin, N-aceylcystein (NAC) was purchased from Sigma Aldrich and dissolved in PBS as a 5 mM stock. Taxol was purchased from Santa Cruz Biotechnology (Santa Cruz, CA, USA) (sc-201439A).

### Cell culture and transfection

The human non–small lung cancer cell line NCI-H460 was cultured in RPMI (Gibco, Gaithersburg, MD, USA) supplemented with 10% fetal bovine serum (Gibco) in a 37°C incubator with 5% CO_2_. Transfections were performed using Lipofectamine 2000 (Invitrogen, Carlsbad, CA USA). Lentiviruses were used to create stable NCI-H460 cell lines expressing shRNA for HSP27 (puromycin resistance gene). The HSP27 shRNA plasmid (sc-29350-SH), and shRNA plasmid transfection reagent (sc-108061) were ordered from Santa Cruz Biotechnology. To generate the sh-Control, and sh-HSP27 cells, the cell lines were transduced with 1 mol of lentivirus and selected using puromycin (1 ug/mL) for at least one week.

### DNA constructs and antibodies

Wildtype (WT) mouse HSP25 (GenBank accession number, NM_013560.2) was polymerase chain reaction (PCR)-amplified, and the dimerization mutant HSP25 (C141A) was constructed by overlap extension PCR. The PCR fragments were digested with BamHI-EcoRI and ligated into p3x-Flag-Myc-CMV-26, yielding p3x-flag-HSP25 (WT) and p3x-flag-HSP25 (C141A). WT and dimerization mutant (C141A) HSP25 were cloned into p3x-Flag-myc-cmv-26 expression vector [21]. Goat polyclonal anti-HSP27 (sc-1049), goat polyclonal anti-HSP70 (sc-1060), mouse monoclonal anti-HSP90α/β (sc-13119), mouse monoclonal anti-HSF1 (sc-17757), rabbit-polyclonal anti-PKCδ (sc-213) and mouse monoclonal anti-β-actin (sc-47778) antibodies were purchased from Santa Cruz Biotechnology. Rabbit polyclonal anti-cleaved caspase-3 (#9661), rabbit polyclonal anti-cleaved PARP (#9541), and rabbit polyclonal anti-cleaved cytochrome c (#9661) antibodies were purchased from Cell Signaling (Beverly, MA, USA). Mouse monoclonal anti-cytochrome c (#556432, BD Biosciences) and rabbit polyclonal anti-PARP (#9542, Cell signaling) antibodies were used for immuneprecipitation. Mouse-monoclonal anti-HSP27 (ADI-SPA-800, Enzo) antibody was also used for immunohistochemistry.

### Polyacrylamide gel electrophoresis and Western blot

For polyacrylamide gel electrophoresis (PAGE) and Western blot (WB) analysis, cells were lysed with RIPA (Radio immunoprecipitation assay) buffer (20 mM Tris-HCl [pH 7.5], 150 mM NaCl, 1 mM EDTA (ethylenediamine tetraacetic acid), 1% NP-40, 1% sodium deoxycholate, 1 mM glycerophosphate, 1mM Na_3_VO_4_, 4 mM NaF) supplemented with protease inhibitor cocktail (Calbiochem, La Jolla, CA, USA). The samples were boiled for 5 min, and an equal amount of protein was analyzed on SDS-PAGE. For non-reducing SDS-PAGE, the reducing agents Dithiothreitol and β-mercaptoethanol were omitted from the samples and directly loaded onto gels without boiling.

### Immunoprecipitation

For immunoprecipitation experiments, cells were lysed in immunoprecipitation buffer (20 mM Tris-HCl [pH 7.5], 150 mM NaCl, 1 mM EDTA (ethylenediamine tetraacetic acid), 1% NP-40, 1% sodium deoxycholate, 1 mM glycerophosphate, 1 mM Na_3_VO_4_, 4 mM NaF). After lysates were centrifuged (30 min at 15,814 × g) to remove particulate material, the supernatant was incubated with primary antibodies with gentle rocking overnight at 4ºC. The immunocomplexes were precipitated with protein A agarose beads and incubated at 4ºC. After 90 min, the samples were analyzed by immunoblotting.

### Irradiation

Cells were exposed to γ -rays with a ^137^Cs γ-ray source (MDS Nordion, Ottawa, Canada) with a dose rate of 2.34 Gy/min.

### Identification of protein band

p3x-Flag-myc-cmv-26 vector was used to express a Flag tag and the full sequence of mouse HSP25. NCI-H460 cells were transfected with the p3x-flag-HSP25 (WT) and p3x-flag-HSP25 (C141A) and treated with ZER (10 μM) for 12 h. Cell lysates were separated by SDS-PAGE. Untreated cells were also used as a control. To estimate the location of a band, a Coomassie Brilliant Blue R-250–stained gel was compared by Western blot analysis. Protein bands were excised and digested with trypsin. The digests were analyzed by quadrupole time-of-flight (Q-TOF).

### MTT analysis

Cell viability was evaluated using MTT assay. For MTT assay, cells (1.5 × 10^5^ cells/ml) were seeded in 96-well plates (Corning Inc., NY, USA) overnight and treated SW15 (10 μM), taxol (0.01 μM) or cisplatin (5 μM) for 24 h at 37°C. After treatment, the cell viability was determined by adding 10 μl MTT solution (5 mg/ml in PBS) to each well followed by incubation for 4 h at 37°C with 5% CO_2_. The MTT mixture was removed and 150 μl DMSO was added to each well. Samples were agitated on a shaker for 30 min, and the absorbance at 540 nm was recorded using a micro-plate reader (Bio-Tek, ELX800, USA). Cell viability was calculated as follows: (1—average absorbance of treated group/average absorbance of control group) × 100%.

### Flow cytometry analysis

The cells were washed twice with 1× phosphate-buffered saline (PBS) and incubated in the dark for 10 min at 37^°^C in PBS containing 10 μg/ml propidium iodide (Sigma Aldrich). Flow cytometric analysis was performed using a FACScan flow cytometer (Becton Dickinson, Franklin Lakes, NJ, USA).

### Real time PCR (RT-PCR)

Total RNA was extracted using QIAzol (QIAGEN). cDNA was synthesized using the ReverTra Ace RT-PCR kit (Toyobo, Osaka, Japan). The transcript levels of *hsp27*, and *hsp70* were measured by RT-PCR (GenDEPOT, Barker, TX, USA). *gapdh* was used as an internal control gene.

### Tumor xenografts in nude mice

A single NCI-H460 cell suspension (1 × 10^6^ cells) was injected subcutaneously into the hind legs of 6-week-old BALB/c athymic nude mice (SLC, Hamamatsu, Japan). The volume injected was 50 μl per mouse to avoid leakage, and a different site was used for each injection. When the tumor reached a minimal volume of 100–300 mm^3^, xenografted mice were treated 6 times with SW15, SW13, or YK594 (6.8 mg/kg) with or without treatment of local regional application of 17-AAG (25 mg/kg) or of treatment of IR (5 Gy) on the whole body. Tumor volumes were determined according to the formula (L × W^2^)/2, by measuring tumor length (L) and width (W) with a caliper. Tumors were measured twice weekly and allowed to grow for 3 weeks.

### TUNEL assay

For the TUNEL assay, the Peroxidase *In Situ* Apoptosis Detection Kit was used according to the manufacturer's recommendations. The number of TUNEL-positive tumor cells and the total number of tumor cells were measured in three microscopic fields of randomly selected tumors and then the mean value was calculated as the percentage of TUNEL-positive tumor cells.

### Immunohistochemistry

Immunohistochemistry was performed on sections of paraffin-embedded specimens using monoclonal anti-Ki-67 antibody (Dako, M7240) or monoclonal anti-HSP27 antibody (Santa Cruz Biotechnology, sc-13132). Briefly, after deparaffinization and hydration, the sections were treated with heat-mediated antigen retrieval using 10 mM citrate buffer (pH 6.0) for 30 min. Then the endogenous peroxidase activity was quenched by 3% hydrogen peroxide solution for 10 min. Non-specific binding was prevented by incubation with 5% normal goat serum for 1 h at 37°C. After that, the sections were incubated with anti-HSP27 antibody (1:250 dilutions) or anti-Ki-67 antibody (1:150 dilutions) as the primary antibody overnight at 4°C in a moist chamber. Secondary antibody incubation and staining were performed using the EnVision^®^+ System–HRP (ABC) kit (Vector Laboratories, Burlingame, CA, USA) according to the manufacturer's recommendations and observed using light microscopy (Carl Zeiss Axio Scope, A1).

### Survival analysis

Survival analyses were performed with on-line survival analysis (*www.kmplot.com*).

### Statistical analysis

Values are displayed as mean plus or minus SEM. Comparisons between groups were carried out by the one-way ANOVA for experiments with more than three subgroups. Post hoc range tests were performed with the one-way ANOVA. Results were considered statistically significant for *P* values less than 0.05.

## SUPPLEMENTARY MATERIAL FIGURES


