# Supplementary material for: Overcoming HSP27-mediated resistance by altered dimerization of HSP27 using small molecules
Source: Oncotarget. 2016 Jul 16;7(33):53178–90. doi: 10.18632/oncotarget.10629 (PMC5288177; doi:10.18632/oncotarget.10629)
Supplement: Supplementary file 1 [file oncotarget-07-53178-s001.pdf]

Overcoming HSP27-mediated resistance by altered dimerization of HSP27 using small molecules

Supplementary Materials

SUPPLEMENTARY MATERIALS AND METHODS

Chemicals synthesis

Most of the chemicals and reagents used were obtained from Aldrich Chemical Co. and Tokyo Chemicals. Melting points were measured without correction in open capillaries with Barnstead Electrothermal melting point apparatus, Manual MEL-TEMP (Model No: 1202D). Chromatographic separations were monitored by thin-layer chromatography using a commercially available pre-coated Merck Kieselgel 60 F_{254} plate (0.25 mm) and detected by visualizing under UV at 254 and 365 nm. Silica gel column chromatography was carried out with Merck Kiesel gel 60 (0.040–0.063 mm). All solvents used for chromatography were directly used without distillation. The purity was assessed by HPLC (Shimadzu LC-6AD) analysis under the following conditions; column, Synergi 4u Fusion-RP 80A (Phenomenex, 4.6 mm i.d. × 250 mm, 4 micron); mobile phase, isocratic elution of methanol; flow rate, 1.0 mL/min; detection, UV detector (Shimadzu SPD-M20A diode array detector, 400 nm). The purity of compound is described as percent (%). NMR spectra were recorded on Varian AS 400 (^{1}H-NMR at 400 MHz and ^{13}C-NMR at 100 MHz) with tetramethyl silane as an internal standard. Chemical shift (δ) values are expressed in ppm and coupling constant (*J*) values in hertz (Hz). Mass spectral investigations were run on electrospray ionization–time of flight mass spectrometry (Agilent) in a positive mode.

1,3-Bis(thiiran-2-ylmethoxy)-9H-xanthen-9-one (SW15)

To a mixture of 1,3-dihydroxy-9H-xanthen-9-one (SW15, 0.50 g, 2.19 mmole) and Cs_2CO_3 (1.43 g, 4.28 mmole) in acetone/DMF (14 mL/7 mL) was added epithiochlorohydrin, (0.71 g, 6.57 mmole). The reaction mixture was stirred at 62°C for 20 h and cooled to room temperature. Water was added and reaction mixture was extracted with ethyl acetate. Organic layer was combined and washed with brine and then dried over anhydrous $MgSO_4$. Solvent was emoved under reduced pressue and the residue was purified by silica gel column chromatography (ethyl acetate:*n*-hexane = 1:2 → 1:1) to give compound 1 as a weak yellow solid (40 mg, 6.1%) and compound 3 as a white solid (148 mg, 18.1%).

SW15: m.p. 150–152°C; R_f 0.68 (ethyl acetate:*n*-hexane = 1:1(v/v)); HPLC: R_T 4.06 min (purity; 99.7%); ^{1}H-NMR (400 MHz, $CDCl_3$) δ 2.36 (dd, *J* = 5.2, 1.2 Hz, 1H), 2.45 (d, *J* = 5.2 Hz, 1H), 2.65 (d , *J* = 6.0 Hz, 1H), 2.71 (d, J = 6.0 Hz, 1H), 3.26–3.32 (m, 1H), 3.44–3.50 (m, 1H), 3.95 (dd, *J* = 10.0, 7.2 Hz, 1 H), 4.03 (dd, *J* = 10.0, 6.8 Hz, 1H), 4.25 (dd, *J* = 10.0, 6.0 Hz, 1H), 4.45 (dd, *J* = 10.0, 4.8 Hz, 1H), 6.36 (d, *J* = 2.4 Hz, 1H), 6.51 (d, *J* = 2.4 Hz, 1H), 7.31-7.38 (m, 2H), 7.64 (ddd, *J* = 7.2, 7.2, 2.0 Hz, 1H), 8.28 (dd, *J* = 8.0, 1.6 Hz, 1H); ^{13}C-NMR (100 MHz, $CDCl_3$) 23.9, 24.7, 30.9, 31.2, 73.1, 74.0, 94.4, 97.4, 108.2, 117.2, 123.2, 124.2, 126.9, 134.1, 155.2, 159.9, 161.0, 163.6, 175.5 ppm; HRMS (ESI) $[M+H]^+$ $C_{19}H_{16}O_4S_2$ calcd 373.0563, found 373.0570.

1-Hydroxy-3-methoxy-9*H*-xanthen-9-one (SW13)

To a mixture of 1,3-dihydroxy-9H-xanthen-9-one, (SW13, 0.50 g, 2.19 mmol) and K_2CO_3 (0.30 g, 2.17 mmol) in acetone/DMF (20 mL/10 mL) was added CH_3I (0.47 g, 3.29 mmol) and the reaction mixture was stirred at room temperature for 20 h. Water was added and solid formedwas filtered and dried to give compound II as a weak yellow solid (388 mg, 73.7%). R_f 0.58 (ethyl acetate:*n*-hexane = 1:3(v/v)); ^{1}H-NMR (400 MHz, $CDCl_3$) δ 3.89 (s, 3H), 6.35 (d, *J* = 2.0 Hz, 1H), 6.43 (d, *J* = 2.0 Hz, 1H), 7.37 (ddd, *J* = 8.0, 7.2, 0.8 Hz, 1H), 7.42 (dd, *J* = 8.0, 0.8 Hz, 1H), 7.71 (ddd, *J* = 7.2, 7.2, 1.6 Hz, 1H), 8.24 (dd, *J* = 8.0, 1.6 Hz, 1H), 12.86 (s, 1H); ^{13}C-NMR (100 MHz, $CDCl_3$) 56.0, 93.0, 97.3, 104.2, 117.8, 120.9, 124.2, 126.1, 135.2, 156.2, 157.9, 163.8, 167.0, 181.0 ppm.

3-Methoxy-1-(thiiran-2-ylmethoxy)-9H-xanthen-9-one (YK594)

A mixture of 1-hydroxy-3-methoxy-9*H*-xanthen-9-one (II) (YK594, 0.30 g, 1.25 mmol) and K_2CO_3 (0.26 g, 1.87 mmol) inaceton/DMF (8 mL/8 mL) was added epithiochlorohydrin, (0.27 g, 2.50 mmol). The reaction mixture was stirred at 64°C for 20 h and cooled to room temperature. Water was added and reaction mixture was extracted with ethyl acetate. Organic layer was combined and washed with brine and then dried over anhydrous $MgSO_4$. Solvent was emoved under reduced pressue and the residue was purified by silica gel column chromatography (ethyl acetate:*n*-hexane = 1:2 → 1:1) to give compound 1 as a weak yellow solid (40 mg, 6.1%)

and compound 5 as a white solid (0.24 g, 59.9%). m.p. 159–160°C; R*f* 0.33 (ethyl acetate:*n*-hexane = 1:3(v/v)); HPLC: R_T 4.01 min (purity; 99.8%); ^{1}H-NMR (400 MHz, $CDCl_3$) δ 2.53 (dd, *J* = 5.2, 1.2 Hz, 1H), 2.71 (dd, *J* = 6.0, 1.2 Hz, 1H), 3.44-3.50 (m, 1H), 3.90 (s, 3H), 3.94 (dd, *J* = 10.4, 3.6 Hz, 1H), 4.45 (dd, *J* = 10.4, 4.0 Hz, 1H), 6.34 (dd, *J* = 2.0 Hz, 1H), 6.53 (d, *J* = 2.0 Hz, 1H), 7.32 (ddd, *J* = 7.2, 7.2, 0.8 Hz, 1H), 7.37 (dd, *J* = 8.0, 0.8 Hz, 1H), 7.63 (ddd, *J* = 7.2, 7.2, 2.0 Hz, 1H), 8.29 (dd, *J* = 8.0, 1.2 Hz, 1H); ^{13}C-NMR (100 MHz, $CDCl_3$) 24.7, 31.2, 56.0, 74.0, 93.9, 97.2, 107.9, 117.2, 123.2, 124.1, 126.9, 134.0, 155.2, 160.0, 160.9, 165.0 ppm; HRMS (ESI) $[M+H]^+$ $C_{17}H_{14}O_4S$ calcd 315.0686, found 315.0686.

Dimer formation of other proteins

NCI-H460 cells were treated with DMSO or SW15 (10 µM) for 24 hr. Cells were lysed with RIPA buffer and subjected to SDS-PAGE. mouse monoclonal anti-HSP90α/β (sc-13119), goat polyclonal anti-HSP70 (sc-1060), Goat polyclonal anti-Akt1 (sc-7126), rabbit-polyclonal anti-NFκB (sc-109), and Anti-β-Actin (sc-47778) antibodies were purchased from Santa Cruz Biotechnology, Inc. Rabbit monoclonal anti-JNK2 (#9258) antibody was purchased from Cell signaling (Beverly, MA, USA).

Identification of protein band

pcDNA3 vector (Invitrogen) was used to express a Flag tag and the full sequence of mouse HSP25. NCI-H460 cells were transfected with the pcDNA3-Flag-HSP25 and treated with SW15 (10 µM) for 48 hr. Cell lysates were separated by SDS-PAGE. Untreated cells were also used as a control. To estimate the location of a band, a Coomassie Brilliant Blue R-250-stained gel was compared by Western blot analysis. Protein bands were excised and digested with trypsin. The digests were analyzed by Q-TOF.

A

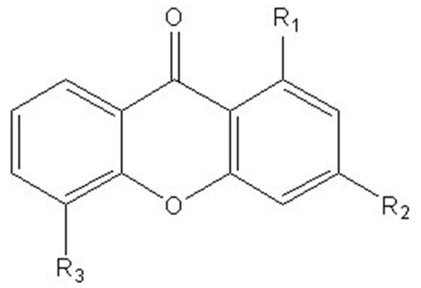

Compound	R1	R2	R3
SW2	OH	$C_3H_5O_2$	H
SW7	$C_3H_5O_2$	$C_3H_5O_2$	H
SW8	$C_3H_5O_2$	$C_3H_5O_2$	OCH_3
SW9	OH	$C_4H_7O_2$	H
SW11	$C_4H_7O_2$	$C_4H_7O_2$	H
SW12	$C_4H_7O_2$	$C_4H_7O_2$	OCH_3
SW13	OH	C_3H_5OS	H
SW14	OH	C_3H_5OS	OCH_3
SW15	C_3H_5OS	C_3H_5OS	H
SW16	C_3H_5OS	C_3H_5OS	OCH_3
SW25	OCH_3	$C_3H_6ClO_2$	H
YK594	C_3H_5OS	OCH_3	H

B

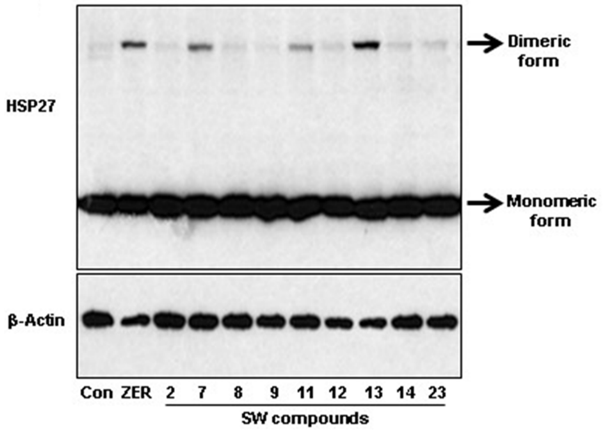

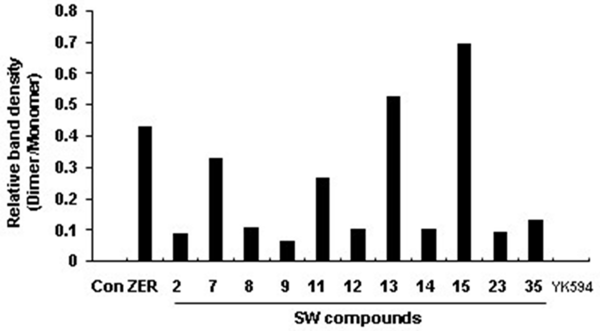

Supplementary Figure S1: The effects of the xanthone compounds on altered cross-linking of HSP27. (**A**) Structures of xanthone compounds. (**B**) NCI-H460 cells were treated with each compound (12.5 µM for 3 h), and cell lysates were analyzed by Western blotting. Zerumbone (ZER, 10 µM) was used for a positive control (left). Relative protein band intensity of dimerized HSP27 was calculated by comparing densitometric scans of the sample immunoblots with the values of control samples set at 1 (right).

Table 1. IC_{50} Values against NCI-H460 human lung cancer cell line

Compounds	IC_{50} (uM)
SW13	> 80
SW15	23.87±1.83
YK594	> 80
Taxol	0.36±0.02

NCI-H460 cells were treated various doses of compounds and after 24 h, flow cytometry analysis after PI staining was performed. Each data point represents mean±SD from three different experiments performed in triplicate.

Supplementary Figure S2: Cellular cytotoxicity SW13, SW15, YK594, 17-AAG, or taxol was applied at the indicated concentrations or radiation doses for 24 h and cell death was analyzed by flow cytometry after PI staining. The table shows the IC_{50} values. Data point represents the mean ± SD.

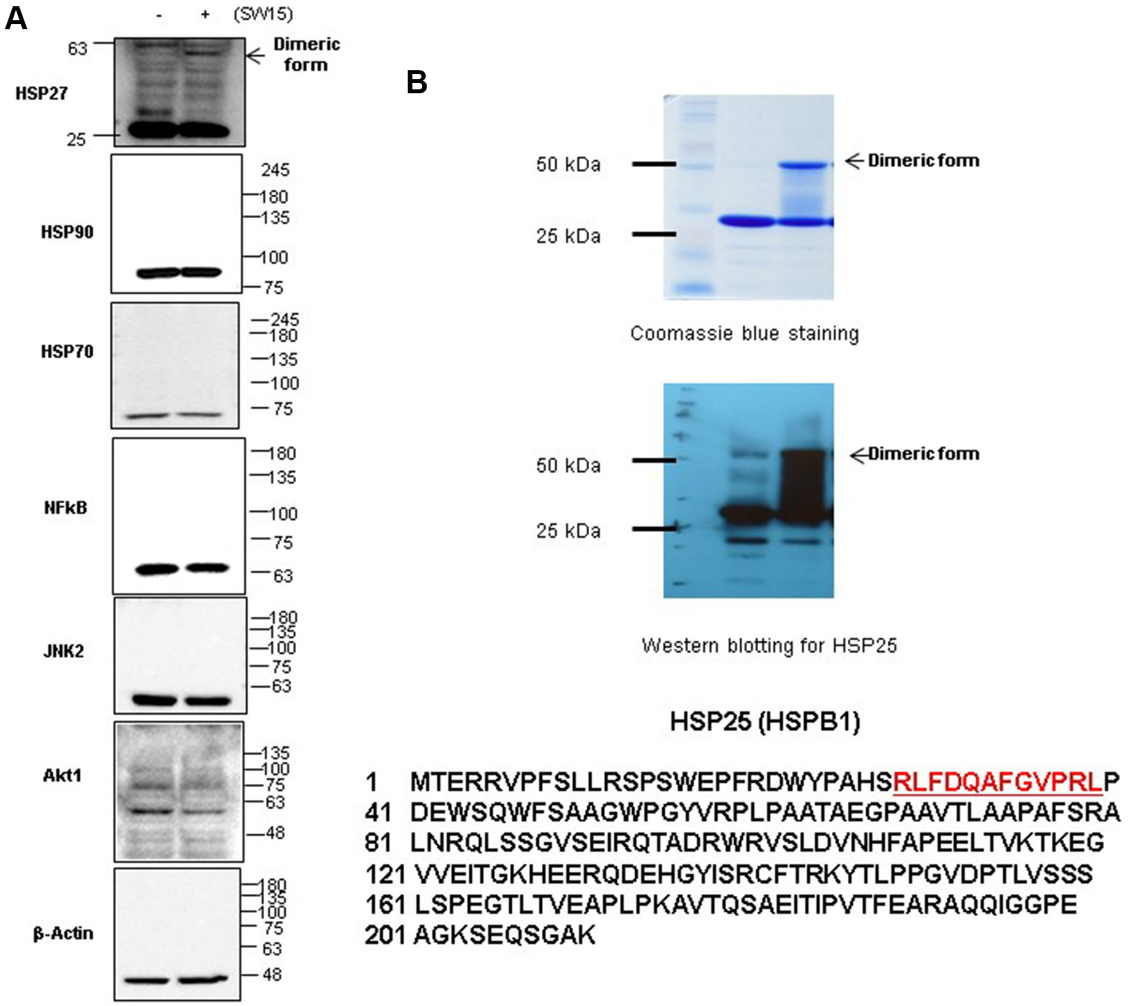

Supplementary Figure S3: HSP27 specific altered dimerization by the xanthone compounds. (**A**) NCI-H460 cells were treated with DMSO or SW15 (10 μM) for 24 h. The cell lysate was subjected to SDS-PAGE and Western blotting. (**B**) NCI-H460 cells were transfected with Flag-HSP27 and treated with DMSO or SW15 (10 μM) for 24 h. The cell lysate was subjected to SDS-PAGE. Flag-HSP25 transfected and SW15-treated cell lysate were also subjected to SDS-PAGE. Non-transfected and DMSO-treated cell lysate was used as a control (Coomasie blue staining gel). Based on Western blot analysis, the band on the gel was excised and analyzed by Q-TOF (upper). Peptides matching the HSP25 protein sequence are underlined (bottom).

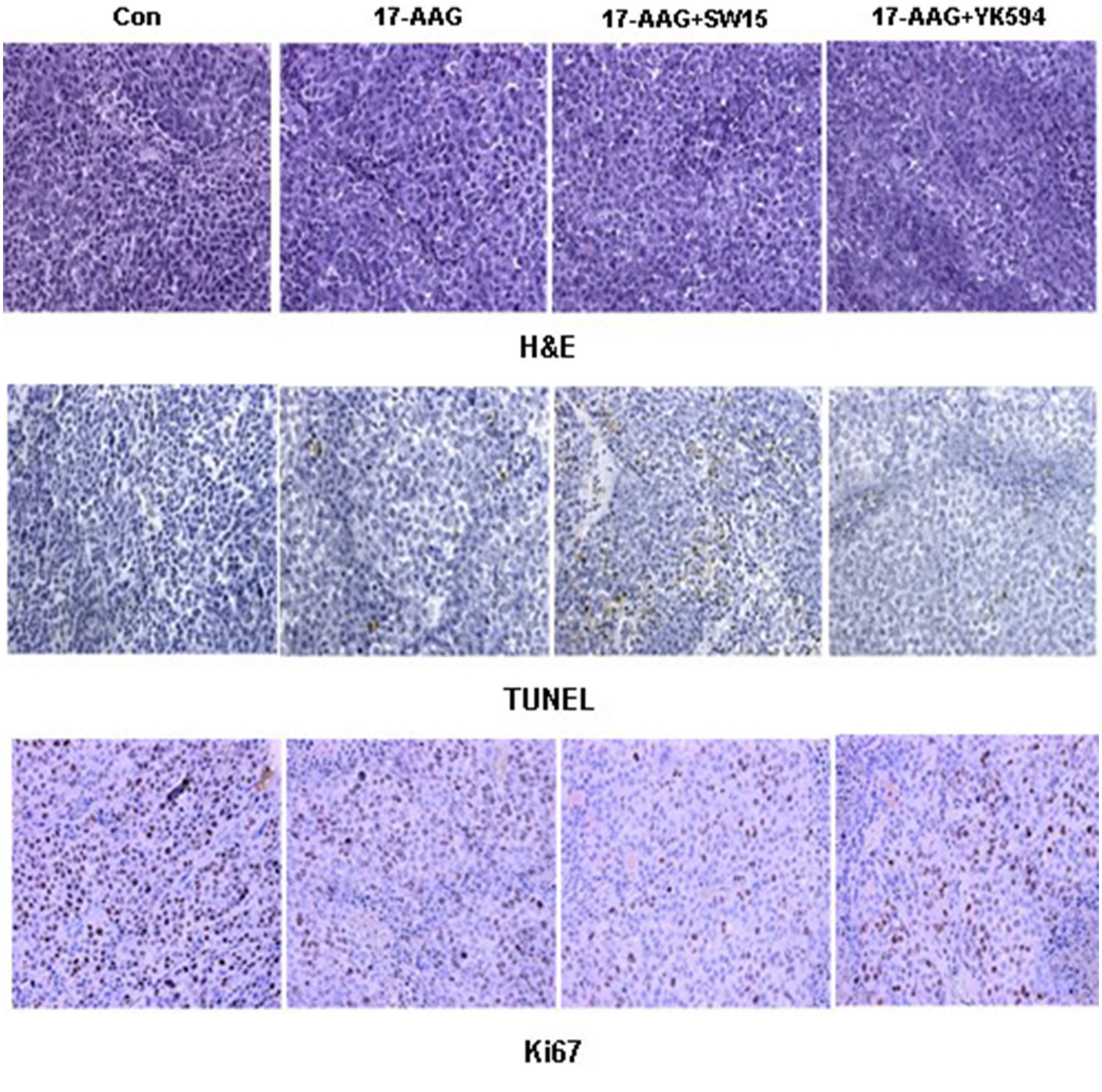

Supplementary Figure S4: H&E staining, TUNEL staining and Ki-67 staining for tumor tissues. NCI-H460 cells were injected subcutaneously into BALB/c nude mice (*n* = 3/group). Xenografted mice were treated 6 times with SW15 or YK594 (6.8 mg/kg per each) with a local regional application in combined with 6 times intraperitoneal treatment of 17-AAG (25 mg/kg). H&E (upper) staining, TUNEL staining (middle) and Ki-67 staining (bottom) were performed using tumor tissues. Representative photos are presented.

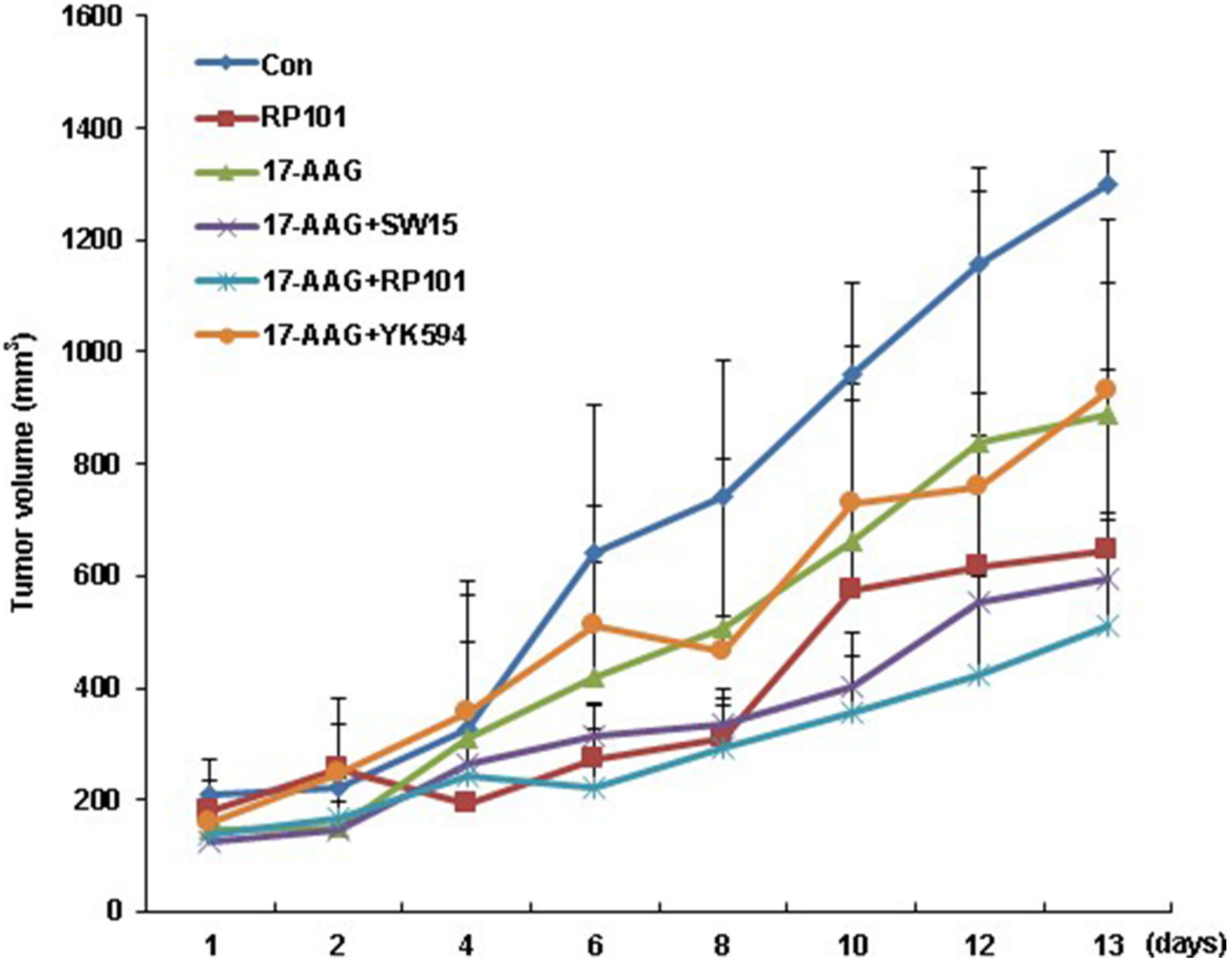

Supplementary Figure S5: Comparison of anticancer activity between SW15 and RP101 in combination with 17-AAG NCI-H460 cells were injected subcutaneously into BALB/c nude mice (*n* = 3/group). Xenografted mice were treated 6 times with SW15 or YK594 (6.8 mg/kg per each) delivered with a local regional application or RP101 (6.8 mg/kg) delivered with intraperitoneal application in combination with 6 times intraperitoneal treatment of 17-AAG (25 mg/kg). Tumor size was measured three times weekly.

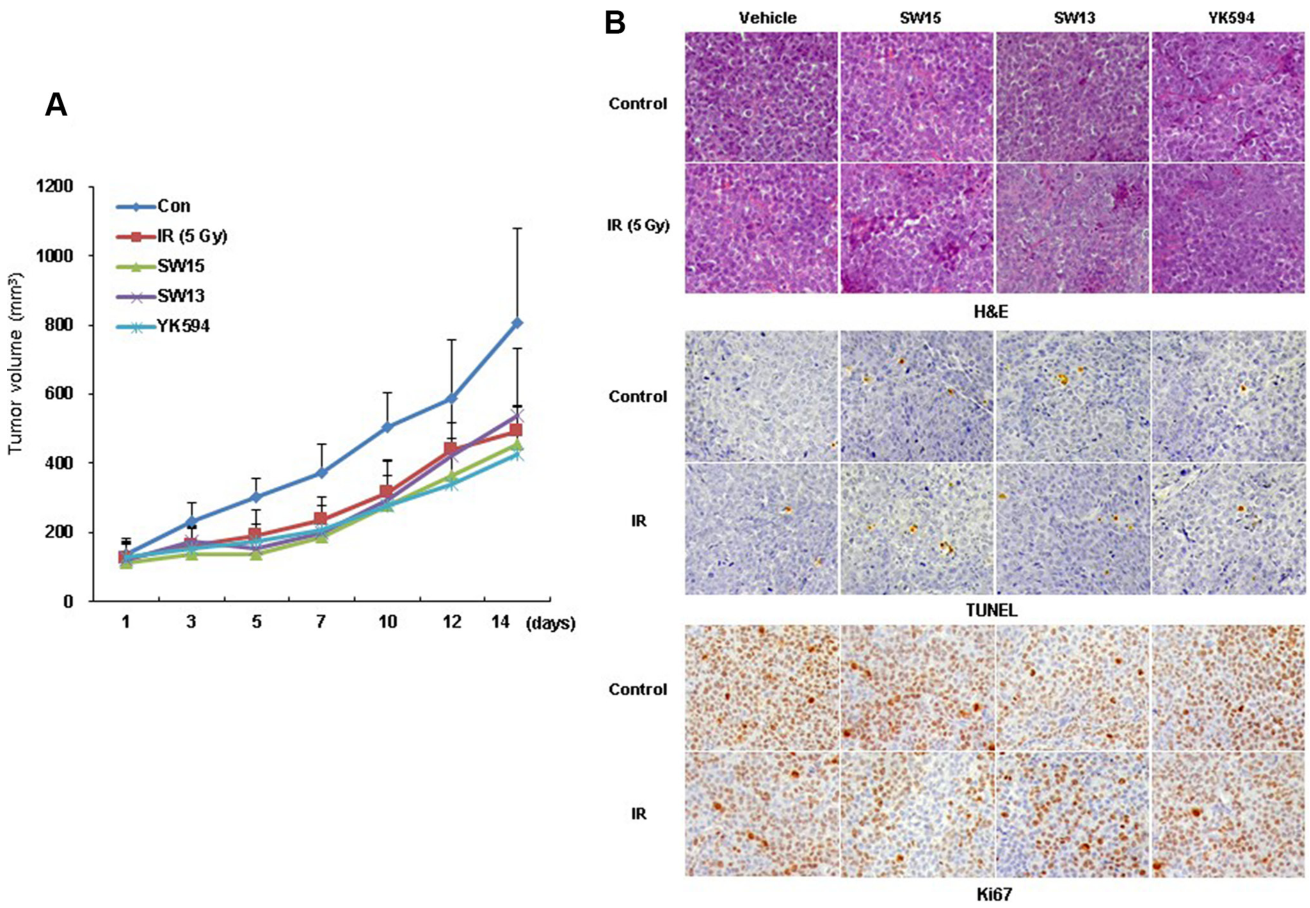

Supplementary Figure S6: Tumor growth after treatment with SW15, SW13, YK594 or IR in a cancer cells xenografted mouse model. (**A**) NCI-H460 cells were injected subcutaneously into BALB/c nude mice (n = 3/group). Xenografted mice were treated 6 times with SW15, SW13, YK594 (6.8 mg/kg) or 5 Gy IR delivered locally. Tumor size was measured twice weekly. (**B**) Representative photos of H&E staining (upper), TUNEL staining (middle) and Ki-67 staining (bottom) in tumor tissues.

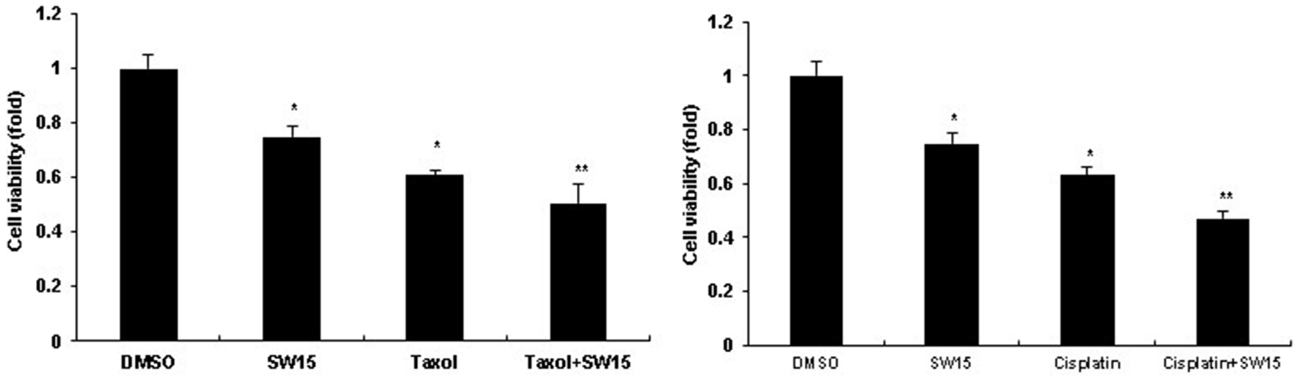

Supplementary Figure S7: The xanthone compound induced sensitization to cancer cells in combination with conventional anticancer drugs. NCI-H460 cells were treated with DMSO or SW15 (10 μM) for 24 h, with or without cisplatin (3 μM) or Taxol (0.01 μM), and cell survival was analyzed by MTT assay. Results are the means and standard deviations of three independent experiments (*$p < 0.05$).
